# Topological nodal-line fermions in spin-orbit metal PbTaSe_2_

**DOI:** 10.1038/ncomms10556

**Published:** 2016-02-02

**Authors:** Guang Bian, Tay-Rong Chang, Raman Sankar, Su-Yang Xu, Hao Zheng, Titus Neupert, Ching-Kai Chiu, Shin-Ming Huang, Guoqing Chang, Ilya Belopolski, Daniel S. Sanchez, Madhab Neupane, Nasser Alidoust, Chang Liu, BaoKai Wang, Chi-Cheng Lee, Horng-Tay Jeng, Chenglong Zhang, Zhujun Yuan, Shuang Jia, Arun Bansil, Fangcheng Chou, Hsin Lin, M. Zahid Hasan

**Affiliations:** 1 Department of Physics, Laboratory for Topological Quantum Matter and Spectroscopy (B7), Princeton University, Princeton, New Jersey 08544, USA; 2 Department of Physics, National Tsing Hua University, Hsinchu 30013, Taiwan; 3 Center for Condensed Matter Sciences, National Taiwan University, Taipei 10617, Taiwan; 4 Princeton Center for Theoretical Science, Princeton University, Princeton, New Jersey 08544, USA; 5 Department of Physics and Astronomy, University of British Columbia, Vancouver, British Columbia, Canada V6T 1Z1; 6 Centre for Advanced 2D Materials and Graphene Research Centre National University of Singapore, 6 Science Drive 2, Singapore 117546, Singapore; 7 Department of Physics, National University of Singapore, 2 Science Drive 3, Singapore 117542, Singapore; 8 Department of Physics, Northeastern University, Boston, Massachusetts 02115, USA; 9 Institute of Physics, Academia Sinica, Taipei 11529, Taiwan; 10 ICQM, School of Physics, Peking University, Beijing 100871, China

## Abstract

Topological semimetals can support one-dimensional Fermi lines or zero-dimensional Weyl points in momentum space, where the valence and conduction bands touch. While the degeneracy points in Weyl semimetals are robust against any perturbation that preserves translational symmetry, nodal lines require protection by additional crystalline symmetries such as mirror reflection. Here we report, based on a systematic theoretical study and a detailed experimental characterization, the existence of topological nodal-line states in the non-centrosymmetric compound PbTaSe_2_ with strong spin-orbit coupling. Remarkably, the spin-orbit nodal lines in PbTaSe_2_ are not only protected by the reflection symmetry but also characterized by an integer topological invariant. Our detailed angle-resolved photoemission measurements, first-principles simulations and theoretical topological analysis illustrate the physical mechanism underlying the formation of the topological nodal-line states and associated surface states for the first time, thus paving the way towards exploring the exotic properties of the topological nodal-line fermions in condensed matter systems.

## Abstract (web summary)

Nodal-line shaped bands appearing near the Fermi level host unique properties in topological matter, which has yet to be confirmed in real materials. Here, the authors report the existence of topological nodal-line states in the non-centrosymmetric single-crystalline spin-orbit semimetal PbTaSe_2_.

## Graphical abstract

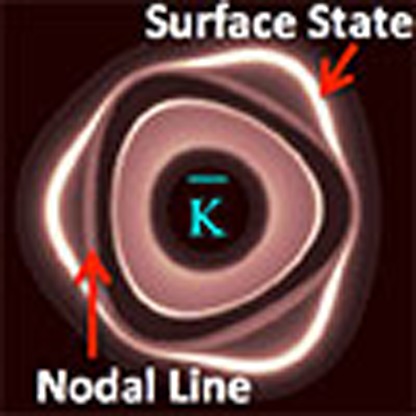

The discovery of the time-reversal invariant topological insulator has stimulated an enormous research interest in novel topological states protected by different symmetries^1^,^2^,^3^. One of the key properties of topological materials is the existence of symmetry-protected metallic edge or surface modes in bulk-insulating ground states, which is due to a topologically nontrivial ordering of bulk wave functions. Recently, because of the experimental observations of Weyl semimetals^4^,^5^,^6^,^7^,^8^,^9^,^10^,^11^,^12^,^13^,^14^, the research interest in topological phenomena in condensed matter systems has partially shifted from insulating materials to semimetals and metals. A Weyl semimetal is a topological state of matter whose low-energy bulk electrons are linearly dispersing Weyl fermions. The twofold degenerate Weyl nodes, carrying non-zero chiral charge, are connected on the boundary by Fermi arc surface states, which are predicted to exhibit unusual transport behaviours such as negative magnetoresistance and non-local transport current^15^,^16^,^17^,^18^. In contrast to Weyl semimetals whose bulk Fermi surface has dimension zero, nodal-line semimetals have extended band touching along one-dimensional curves in *k* space, presenting a significant expansion of topological materials beyond topological insulators and Weyl semimetals, and new opportunities to explore exotic topological nodal physics. Line-like touchings of a conduction and valence band need extra symmetries besides translation, such as mirror reflection, to be topologically protected. Kinematically, this protection involves a finite fraction of Brillouin zone. For this reason, this leads potentially to many anomalies in electromagnetic and transport response^19^,^20^,^21^,^22^. Similar to the case of Weyl nodes, one can define an integer topological invariant for the line node along which two nondegenerate bands touch^19^. Despite the many theoretical discussions of nodal-line semimetals, a material realization of topological nodal-line fermions has been lacking for many years, just like Weyl semimetals.

In this work we performed systematic theoretical study and experimental characterization of the electronic structure of a spin-orbit metal PbTaSe_2_, indicating the existence of topological nodal-line phase in this compound for the first time. The crystal lattice of this material lacks space inversion symmetry, which lifts the spin degeneracy of its electronic bulk bands. Our angle-resolved photoemission (ARPES) measurements together with density functional theory (DFT) calculations show that the conduction band originated from Pb-6*p* orbitals and the valence band from Ta-5*d* orbitals cross each other, forming three nodal-line states close to the Fermi energy. The nodal lines are protected by a reflection symmetry of the space group. The topological nodal-line state in PbTaSe_2_ belongs to the symmetry class A+*R* (*p*=2) of symmetry-protected semimetals^19^. We also demonstrate through effective Hamiltonian modelling and DFT simulations that the nodal lines are accompanied by surface bands. These surface states are due to the *π* Berry phase agglomerated around the nodal line in analogy to the states on the graphene zigzag edge. Our detailed theoretical modelling and calculation, aided by a systematic experimental characterization, establish the existence of topological nodal-line fermions in the superconducting compound PbTaSe_2_, opening the door for exploring the exotic properties of nodal-line states in condensed matter.

## Results

### Crystal and electronic structure

**Figure 1:**
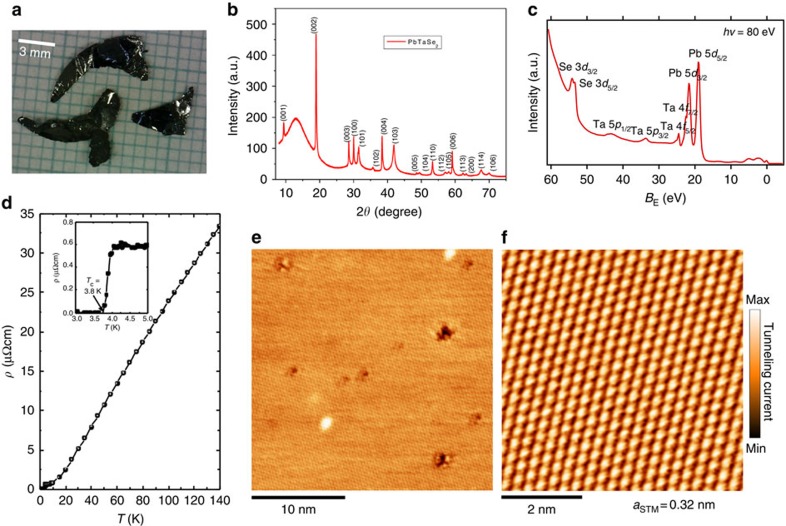
Overview of PbTaSe_2_ single crystal. (**a**) Optical image of PbTaSe_2_ single crystals measured in this work. (**b**) X-ray diffraction measurements showing the lattice parameters matching with the space group (187) 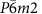. (**c**) ARPES core-level spectrum showing clear Pb-5*d*, Se-3*d* and Ta-4*f* core-level peaks. (**d**) Resistivity as a function of temperature showing a superconducting transition at 3.8 K. (**e**,**f**) STM topography of PbTaSe_2_, indicative of a surface lattice constant 3.2 Å.

Our PbTaSe_2_ single crystals were prepared by the chemical vapour transport method, see Fig. 1a. The samples were of high structural quality, which was confirmed by our X-ray diffraction and scanning tunnelling microscopy (STM) measurements. The X-ray diffraction peaks shown in Fig. 1b are consistent with the space group of PbTaSe_2_, 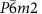 (187), therefore demonstrating the lack of inversion symmetry of our PbTaSe_2_ single crystals. This is crucially important for lifting the spin degeneracy, a necessary condition for the formation of topological nodal lines. To further check the chemical composition of our samples, we performed a photoemission core-level scan. Clear Pb-5*d*, Ta-4*f* and Se-3*d* core-level peaks were observed in the photoemission spectrum, which confirms the correct chemical composition in our PbTaSe_2_ single-crystal samples, shown in Fig. 1c. To verify the superconducting property of our samples, a transport measurement was carried out. The measured resistivity curve (Fig. 1d) shows a clear superconducting transition temperature at 3.8 K, consistent with the value reported in ref. 23. Figure 1e,f shows STM images of the cleaved (001) surface. The topography image clearly reveals a hexagonal lattice with few defects, demonstrating the high quality of our samples. Furthermore, no surface reconstruction was observed on the cleaved surface. The high-resolution STM topography yields a lattice constant of 3.2 Å.

**Figure 2:**
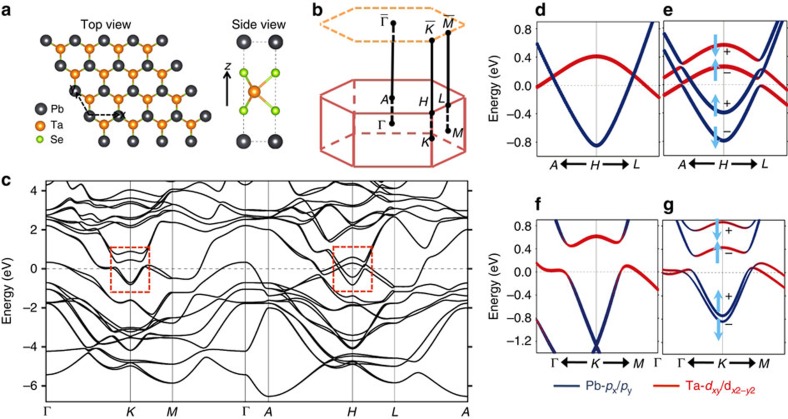
Lattice structure and bulk bands of PbTaSe_2_. (**a**) Hexagonal lattice of PbTaSe_2_. (**b**) Bulk band structure of PbTaSe_2_. (**c**) Calculated bulk band structure of PbTaSe_2_. (**d**,**e**) Zoom-in band structure around *H* without/with the inclusion of SOC. The colour code (red and blue) shows the orbital components. The up and down arrows indicate spin up and spin down along the *z* axis, respectively. (**f**,**g**) Same as **d**,**e** but for band structure around *K*. The bands in **e**,**g** correspond to those in the red rectangular boxes in **c**.

PbTaSe_2_ crystalizes in a hexagonal lattice system in which the unit cell consists of one Pb, one Ta and two Se atoms, and each atom resides on a hexagonal flat layer, shown in Fig. 2a. The stacking sequence of these atomic planes within the unit cell is Pb-Se-Ta-Se: A-A-B-A (A, B and C, here refer to the three high-symmetry spots on a hexagonal lattice). The lattice can also be viewed as a Pb layer intercalating two adjacent TaSe_2_ layers with Pb atoms sitting above Se atoms. The Pb intercalation suppresses the softening of phonon modes associated with the charge density wave in TaSe_2_ and stabilizes the hexagonal lattice on the surface^24^. This particular stacking does not preserve the space inversion symmetry; however, the lattice is reflection-symmetric with respect to the Ta atomic plane. In other words, the Ta atomic planes are a mirror plane of the crystal lattice under the mirror operation *R*_*z*_ that sends *z* to −*z*. This reflection symmetry of the lattice provides a protection for the topological nodal lines, as discussed later on. The bulk and (001)-projected surface Brillouin zones are shown in Fig. 2b. The *A*, *H* and *L* points are high-symmetry points on the *k*_*z*_=*π* plane, which is a mirror plane of the bulk Brillouin zone. Figure 2c presents an overview of the band structure calculation for PbTaSe_2_, which was performed by the method of generalized gradient approximation. Close to the Fermi level, two prominent features in the band structure are observed. One is a giant hole pocket around Γ, whose states are mainly derived from the Ta 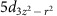 orbitals that are oriented out of the Ta atomic plane, taking the Ta plane as the *x*–*y* plane. The second major contribution to the density of states at the Fermi level comes from the four bands that cross each other near *H*. The two electron-like conduction bands originate from Pb-6*p*_*x*_/*p*_*y*_ orbitals and the two hole-like valence bands from 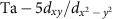 orbitals. We note that all these orbitals are invariant under *R*_*z*_. A zoom-in view of the bands around *H* without/with spin-orbit coupling (SOC) is shown in Fig. 2d,e, respectively. Without the inclusion of SOC, the conduction and valence bands become spin-degenerate. The two bands belong to different representations of the space group (the representation of the electron-like band is A′ and that of the hole-like band is A′′); therefore, the intersection of the two bands is protected by the crystalline symmetry, forming a spinless nodal ring. Once SOC is turned on, each band split into two spin branches with opposite spin orientations and mirror reflection eigenvalues as indicated in Fig. 2e. Only the crossings of branches with opposite mirror reflection eigenvalues remain gapless as a result of symmetry protection, forming a pair of nodal rings. Interestingly, SOC also gives rise to a third nodal ring on *k*_*z*_=0 plane. The detailed band dispersion and the rise of three nodal rings are very well captured by our effective ***k***·***p*** Hamiltonian (Supplementary Note 1 and Supplementary Fig. 1). Before proceeding to a detailed discussion of the nodal-line states, we will present the results of our ARPES measurement, verifying the overall band dispersion of Pb conduction bands and Ta valence bands obtained from our DFT calculation.

### ARPES and DFT results of PbTaSe_2_

**Figure 3:**
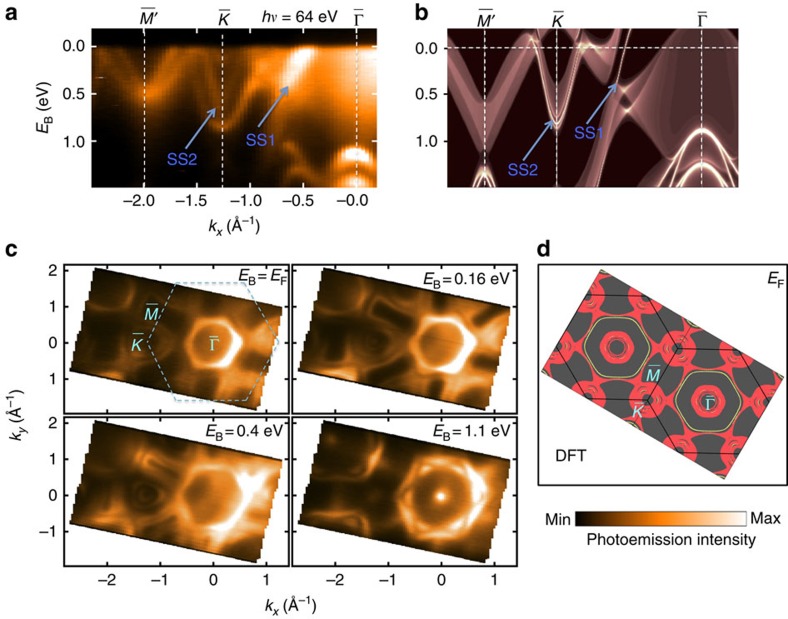
APRES mapping and band calculation of PbTaSe_2_. (**a**) ARPES spectra taken along 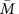
−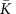
−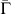 with 64-eV photons. (**b**) DFT-projected bulk bands and surface bands (bright white lines) of (001) surface with Pb termination. (**c**) ARPES isoenergy concours taken with 64-eV photons. (**d**) DFT Fermi surface contour of PbTaSe_2_ (001) surface. The yellow lines indicate the surface states on Pb-terminated (001) surface.

Figure 3a shows a brief overview of our ARPES band mapping, and the corresponding numerical calculation of the PbTaSe_2_ band structure is presented in Fig. 3b. The projected bulk bands and surface bands (as highlighted by white lines) were calculated for the Pb-terminated (001) surface. The DFT band structure reproduces the ARPES spectrum very well. Specifically, in the ARPES spectral cut, a band marked as SS_1_ with high intensity poke the Fermi level between 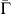 and 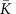. This is the surface band associated with the Pb-terminated (001) surface and it corresponds to Dirac surface mode arising from a continuous bulk band gap opened by SOC^23^,^24^. Around 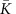 there are three concave bands whose binding energies at 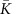 are 0.21, 0.75 and 0.80 eV. The top and bottom bands correspond to the electron-like bands derived from Pb-6*p* orbitals. The middle band, marked as SS_2_, is consistent with the surface band as plotted in Fig. 3b. The two bands at 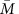 are tails of the two Ta-5*d* bands that cross the two Pb-6*p* bands forming the nodal rings in the vicinity of 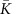. Two Ta-5*d* bands have to degenerate in energy at 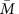 according to the Kramers theorem. The ARPES measured (001) Fermi surface with the incident photon energy of 64 eV, and the theoretical simulations are shown in Fig. 3c,d. At the Fermi level, our data show that the Fermi surface consists of three parts: a hexagon-shaped pocket centred at 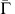 with smeared intensity inside, a dog-bone-shaped contour centred at the 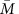 point and several circles surrounding the 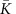 point. Our ARPES data and calculation show an agreement on those features. Furthermore, the hexagon centred at 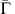 and the intensity inside are the surface band and the bulk hole pocket at 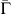, respectively. The dog-bone-shaped contour corresponds to the one branch of the Ta valence band and the circles around 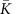 are from the other branch of the Ta valence band; the surface states and the spin-split conduction band are derived from Pb orbitals. As the binding energy decreases, we find that the Pb pockets at 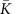 shrink while the Ta pockets expand outwards, which is in good accordance with the characteristics of the electron-like Pb bands and hole-like Ta bands.

**Figure 4:**
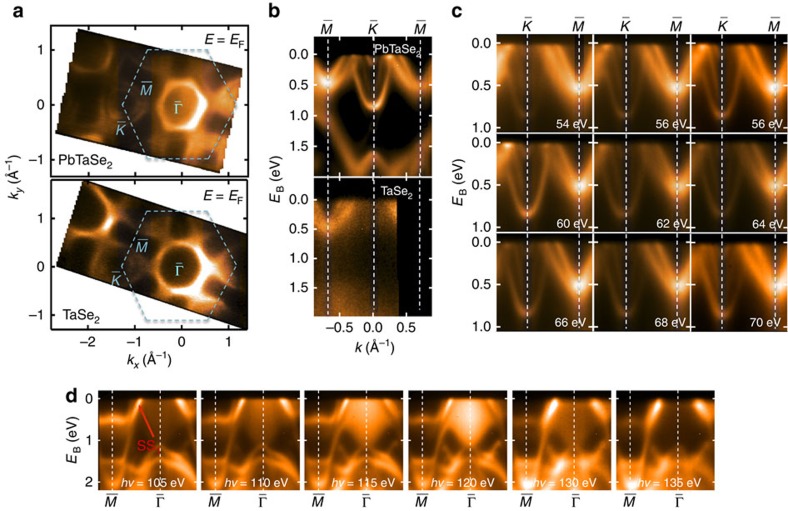
APPES measurement of PbTaSe_2_ and TaSe_2_. (**a**) Fermi surface contour of PbTaSe_2_ and TaSe_2_. (**b**) APRES spectral cut along 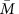
−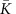
−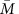 of PbTaSe_2_ (top) and TaSe_2_ (bottom). (**c**) ARPES spectra of PbTaSe_2_ along 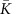
−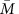 taken with different photon energies. (**d**) ARPES spectra along 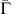
−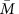 taken with different photon energies.

TaSe_2_ can be regarded as a building block of PbTaSe_2_, and therefore its electronic structure can be traced from that of PbTaSe_2_. To highlight the difference between electronic structures of TaSe_2_ and PbTaSe_2_, we mapped out the Fermi surface and band structure along 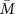
−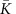
−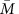 of the two compounds, shown in Fig. 4a,b. In the Fermi surface mapping of TaSe_2_, there are one dog-bone-shaped contour centred at 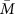 and only one circle-shaped contour centred at 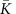. Those contours are from Ta valence bands, and are consistent with previous work^25^. By contrast, the Fermi surface of PbTaSe_2_ has more ring-shaped contours centred at 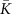, signifying the contribution from the Pb layers. It is easier to view this difference from the 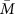
−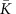
−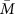 cut. TaSe_2_ does not show any electron-like bands at 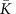 that exist in PbTaSe_2_. Figure 4c shows the ARPES mapping of the Pb and Ta bands of PbTaSe_2_ with photon energies from 54 to 70 eV. The middle band at 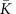 does not show any photon-energy dependence, which is consistent with the surface nature of this band. However, the other bands at 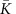 and 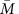 do not exhibit obvious changes with different photon energies either. This seems to contradict to the assignment of those bands as bulk bands according to our DFT calculations. The inconsistency can be understood by considering the fact that the Pb-6*p*_*x*_/*p*_*y*_ and 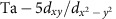 orbitals that constitute those bands are primarily confined within the Pb and Ta atomic planes (which are parallel to the *x*–*y* plane), and, thus, the interlayer couplings (say, the coupling of one Pb-6*p*_*x*_/*p*_*y*_ orbital with another orbital on the adjacent Pb plane) is largely suppressed, which results in little *k*_*z*_/photon-energy dependence. In addition, we mapped out the ARPES spectrum of PbTaSe_2_ along 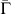
−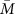 with photon energy from 105 to 135 eV, shown in Fig. 4d. We observed that the intensity from the hole-like bulk pocket around Γ varied prominently with photon energy. It can be attributed to the fact that the states on the hole pocket are mainly derived from the Ta 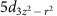 orbitals that are oriented out of the Ta atomic plane and, thus, exhibit strong dispersion along the *k*_*z*_ direction. By contrast, the band marked as SS_1_ did not show any photon-energy dependence, indicative of the surface nature of this band. We note that both our theoretical calculation and our ARPES measurement unambiguously indicate the existence of the electron-like Pb-6*p* bands around 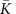, which inevitably cross the hole-like Ta-5*d* bands with a similar energy, leading to the formation of the nodal rings. The remarkable consistency between ARPES result and the our first-principles calculation lay a solid foundation for our theoretical investigation of the topological nodal lines in PbTaSe_2_.

### Nodal lines and drumhead surface states of PbTaSe_2_

**Figure 5:**
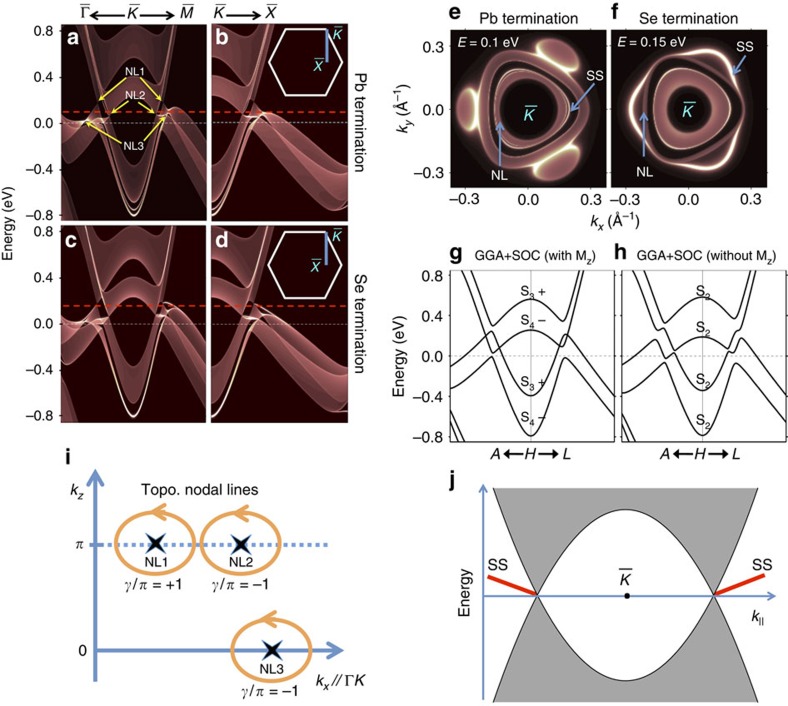
Topological-nodal rings and associated surface states. (**a**,**b**) DFT project bulk bands and surface bands (bright white lines) of Pb-terminated (001) surface along 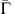
−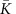
−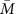 and a generic direction 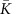
−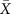, respectively. (**c**,**d**) Same as **a**,**b** but of Se-terminated (001) surface. (**e**,**f**) The isoenergy contour showing the nodal-line states (NL) and surface states (SS). The energy is 0.10 and 0.15 eV above the Fermi level for Pb- and Se termination, respectively, as indicated by the red dashed lines in **a–d**. (**g**,**h**) Bulk band structure of PbTaSe_2_ with and without the reflection symmetry, respectively. In **h**, the reflection symmetry is broken by moving the Pb atom slightly in the vertical direction. (**i**) Schematic of the closed contours encircling the nodal lines for the calculation of the winding number. The arrows indicate the direction on the loops along which the Berry's connection is integrated. (**j**) Schematic of a spectral cut passing 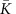. The grey shaded region indicates the projected bulk band associated with a single nodal ring encircling 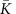, and the red curves depict the surface states (SS).

From the discussion before, we know that the electron-like bands from the intercalated Pb layers are the essential components for forming the topological-nodal-ring band structure. By comparing with the TaSe_2_ spectrum, our ARPES established unambiguously the existence of the Pb bands. To further examine the topological nodal-line states and associated surface states, we calculated the band structure for Pb- and Se-terminated surfaces as shown in Fig. 5a–d. The projected bulk band on each cut from 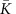 shows three nodal points at 0.05, 0.15 and 0.03 eV above the Fermi level. The first two closer to 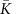 lie on the *k*_*z*_=*π* plane, while the third one is on the *k*_*z*_=0 plane. Let us refer to these three nodal lines as NL1, NL2 and NL3. Corresponding nodal points can be found on a cut of arbitrary orientation that includes 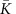. For example, the band structure along a generic direction 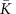
−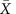 is shown in Fig. 5b,d. Unlike the projected bulk band, which is independent of surface termination, the dispersion of surface bands is found to be sensitive to the surface condition. However, in both cases we do find a surface band connecting to each nodal line, indicative of the topological nature of the bulk nodal lines. In the Pb-termination case, the surface bands disperse outwards with respect to 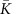, from NL1. The surface band connecting to NL2 grazes inwards at the edge of the lower bulk Dirac cone and merge into the bulk band. The surface band from NL3 disperses inwards with respect to 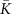, consistent with the SS_2_ band in our ARPES spectrum in Fig. 3, which forms a drumhead surface state contour. By contrast, on the Se-terminated surface the surface band connecting to NL2 first moves into the bulk band gap and then fall into the bulk band region. The surface band from NL1 disperses outwards and connects to NL3. Please refer to Supplementary Fig. 2 and Supplementary Note 2 for a detailed visualization of connection of the surface bands to the bulk nodal lines. To get an overall view of the nodal ring and surface band, we plot in Fig. 5e,f the isoenergy contour in the vicinity of the NL1 nodal ring of the Pb-terminated surface and the NL2 nodal ring of the Se-terminated surface, as indicated by the red dashed lines in Fig. 5a–d. Indeed, gapless nodal points and surface states can be found at every in-plane angle departing from 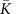. These nodal rings are protected against gap opening by the crystalline symmetry. Specifically, the states in the two Pb branches belong to two different representations of the space group, namely S_3_ and S_4_ as shown in Fig. 5g. The same is true for the two Se branches. In particular, with respect to the Ta atomic plane, the two representations have opposite mirror eigenvalues under the reflection operation. Therefore, gap opening is forbidden at the crossing point between two branches of different mirror eigenvalues, which results in the nodal rings discussed in this work. In this sense, the nodal rings are under the protection of the reflection symmetry. If we shift the Pb atom slightly in the vertical direction, thus breaking the reflection symmetry, all of the four branches are found to belong to the same *S*_2_ representation of the reduced space group and, in this case, a gap opening is allowed at every crossing point of these branches as illustrated in Fig. 5h. A similar gap opening is also found in NL3 on *k*_*z*_=0 plane on breaking the reflection symmetry, please see Supplementary Fig. 3 and Supplementary Note 3.

## Discussion

Let us briefly discuss the topological characterization of the nodal lines and the origin of the surface bands. The material PbTaSe_2_ is time-reversal-symmetric, with time-reversal symmetry represented by 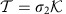, where 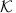 denotes complex conjugation and *σ*_2_ is the second Pauli matrix acting on the electron spin. The mirror symmetry *R*_*z*_ acts in spin space as i*σ*_3_ and therefore commutes with 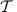. This would place PbTaSe_2_ in class AII in the classification of ref. 19. However, since the nodal lines are centred around momenta *H*/*H*′ and *K*/*K*′, which are not invariant under time-reversal, but pairwise map into each other, the time-reversal symmetry imposes no constraints on the nodal lines individually. The material has, therefore, to be classified according to the time-reversal breaking class A-*R,* which admits an integer topological classification for Fermi surfaces of codimension 2, that is, lines (*p*=2 in ref. 19). The nodal lines carry a topological quantum number *n*^+^, which is given by the difference in the number of occupied bands with *R*_*z*_ eigenvalue +*i* inside and outside the line. In the case at hand, *n*^+^=−1 for the nodal line (NL3) in the *k*_*z*_=0 plane, while *n*^+^=+1 (NL1) and *n*^+^=−1 (NL2) for the two nodal lines in the *k*_*z*_=*π* plane. We have also computed numerically under the DFT framework the winding number 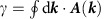, where ***A***(***k***)=i∑_*a*_〈*u*_*a*,***k***_|**∇***u*_*a*,***k***_〉 is the Berry connection of the occupied Bloch bands |*u*_*a*,***k***_〉. For a closed loop encircling any of the nodal lines, we find that *γ*=±*π*, indicating the topological protection of the nodal line as shown in Fig. 5i.

The topological origin of the observed surface states is rather subtle. Surface states associated with the topological invariant *n*^+^ via the bulk-boundary correspondence should only appear on surfaces that preserve *R*_*z*_. The (001) surface, however, breaks *R*_*z*_. The reason why we still observe surface states can be understood from the Berry phase of *π* around the nodal line and the analogy to the edge states on the zigzag edge of graphene. Consider the bulk Hamiltonian on a plane in momentum space that contains both *K* and *H*. At low energies, each nodal ring pierces this plane twice, giving rise to two Dirac cones in this Hamiltonian. These two cones have a Berry phase +*π* and −*π* with respect to the orientation of the plane, precisely as in (spinless) graphene, as schematically depicted in Fig. 5j. We know that for any termination of a graphene sample, an edge state emanates from the projection of each of the Dirac points in the edge Brillouin zone (except for the pathological case where both Dirac points project on the same spot in the edge Brillouin zone). By this analogy, we also expect these edge states to emanate from the surface projections of the nodal lines in any direction away from the 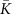 point, thereby forming a surface band. We note that the dispersion of this band and even whether it appears inside or outside the projection of the nodal line is not universal and depends on the details of the surface termination.

Recently, some preprint theoretical lines of work have reported on Ca_3_P_2_ and Cu_3_PdN, proposing that there may be nodal-line states^26^,^27^,^28^. We note that our work is distinct from those lines of work in many ways. Both Ca_3_P_2_ and Cu_3_PdN are centrosymmetric and, therefore, because of the coexistence of time-reversal and inversion symmetry, have fourfold degeneracy at the nodal ring. By contrast, the degeneracy of nodal-ring states is two in PbTaSe_2_ because of the lack of inversion symmetry. In Ca_3_P_2_ and Cu_3_PdN, the nodal-line states exist only in the absence of SOC. In real materials SOC, however, always exists. In PbTaSe_2_, SOC is an essential ingredient for the formation of nodal-ring states. Our ARPES measurement established an experimental characterization of the topological nodal-line material PbTaSe_2_.

In summary, our direct experimental observation by ARPES of the coexistence of Pb concave bands and Ta convex bands centred at 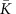 in the non-centrosymmetric superconductor PbTaSe_2_ is in good agreement with our first-principles band structure calculations, establishing the realization of the unusual ring-shaped topological nodal-line states in this compound. The topological nodal rings are protected by the reflection symmetry of the system. Meanwhile, the nodal rings are uniquely associated with drumhead-like surface states in a manner that resembles the connections of edge states and the nodal points in graphene. Considering the one-dimensional nodal characteristics of the bulk band and the two-dimensional topological ‘drumhead' surface states, topological nodal-line semimetals stand as a distinct class of topological materials beyond Weyl semimetals and topological insulators. For example, nodal-line states possess an extra degree of freedom for manipulating novel properties of Weyl materials, which is the finite size of the nodal line. Furthermore, interaction-induced instabilities that have been broadly discussed for Weyl semimetals should be more likely occurring in nodal-line states because of the higher density of states at the Fermi energy. In addition, superconductivity is induced by intercalating Pb layers to TaSe_2_, which also offers the essential ingredient, the Pb-conducting orbitals, for the formation of the topological nodal-line states. Considering the intrinsic superconductivity, the spin-split bulk nodal-line band structure and the nontrivial surface states close to the Fermi level, it is possible that helical superconductivity and *p*-wave Cooper pairing may exist in this compound without the aid of the proximity effect^29^,^30^. Therefore, novel physics may arise from the interplay of the nodal-line states and the emergent superconductivity of PbTaSe_2_, which calls for further experimental investigation on PbTaSe_2_. Our ARPES measurements, detailed DFT simulation and theoretical analysis demonstrate the fundamental mechanism for realizing topological nodal-line fermions in PbTaSe_2_, and pave the way for exploring the exotic properties of topological nodal-line states in condensed matter systems.

## Methods

### Sample growth method

Single crystals of PbTaSe_2_ were grown with the chemical vapor transport (CVT) method using chlorine in the form of PbCl_2_ as a transport agent. For the pure synthesis of PbTaSe_2_, stoichiometric amounts of the elements (purity of Pb and Ta: 6N, of Se: 5N) were loaded into a quartz ampoule, which was evacuated, sealed and fed into a furnace (850 °C) for 5 days. About 10 g of the prereacted PbTaSe_2_ were placed together with a variable amount of PbCl_2_ (purity 5 N) at one end of another silica ampoule (length 30–35 cm, inner diameter 2.0 cm and outer diameter 2.5 mm). All treatments were carried out in an Argon box, with continuous purification of the Argon atmosphere resulting in an oxygen and water content of less than 1 p.p.m. Again, the ampoule was evacuated, sealed and fed into a furnace. The end of the ampoule containing the prereacted material was held at 850 °C, while the crystals grew at the other end of the ampoule at a temperature of 800 °C (corresponding to a temperature gradient of 2.5 K cm^−1^) during a time of typically 1 week. Compact single crystals of sizes of up to 8 × 5 × 5 mm^3^ were obtained.

### ARPES and STM methods

ARPES measurements were performed at the liquid nitrogen temperature in the beamline I4 at the MAX-lab in Lund, Sweden. The energy and momentum resolution was better than 20 meV and 1% of the surface Brillouin zone for ARPES measurements at the beamline I4 at the MAX-lab. Samples were cleaved *in situ* under a vacuum condition better than 1 × 10^−10^ torr. Samples were found to be stable and without degradation for a typical measurement period of 24 h. STM experiments were conducted with a commercial system (Unisoku). Samples were cleaved at room temperature in a vucuum better than 2 × 10^−10^ mbar and were transferred to a STM head precooled to 77 K. Constant-current mode STM images were taken with chemical-etched Pt/Ir tips. Bias voltages were applied to the samples.

### Computational method

We computed electronic structures using the norm-conserving pseudopotentials as implemented in the OpenMX package within the generalized gradient approximation schemes^31^,^32^. Experimental lattice constants were used^33^. A 12 × 12 × 4 Monkhorst-Pack k-point mesh was used in the computations. The SOC effects are included self-consistently^34^. For each Pb atom, three, three, three and two optimized radial functions were allocated for the *s*, *p*, *d* and *f* orbitals (*s*3*p*3*d*3*f*2), respectively, with a cutoff radius of 8 Bohr. For each Ta atom, *d*3*p*2*d*2*f*1 was adopted with a cutoff radius of 7 Bohr. For each Se atom, *d*3*p*2*d*2*f*1 was adopted with a cutoff radius of 7 Bohr. A regular mesh of 300 Ry in real space was used for the numerical integrations and for the solution of the Poisson equation. To calculate the surface electronic structures, we constructed first-principles tight-binding model Hamilton. The tight-binding model matrix elements are calculated by projecting onto the Wannier orbitals^35^. We use Pb *p*, Ta *s* and *d*, and Se *p* orbitals were constructed without performing the procedure for maximizing localization.

## Additional information

**How to cite this article:** Bian, G. *et al.* Topological nodal-line fermions in spin-orbit metal PbTaSe_2_. *Nat. Commun.* 7:10556 doi: 10.1038/ncomms10556 (2016).

## Supplementary Material

Supplementary Information Supplementary Figures 1-3 and Supplementary Notes 1-3
