# Supplementary material for: Topological nodal-line fermions in spin-orbit metal PbTaSe2
Source: Nat Commun. 2016 Feb 2;7:10556. doi: 10.1038/ncomms10556 (PMC4740879; doi:10.1038/ncomms10556)
Supplement: Supplementary Information — Supplementary Figures 1-3 and Supplementary Notes 1-3 [file ncomms10556-s1.pdf]

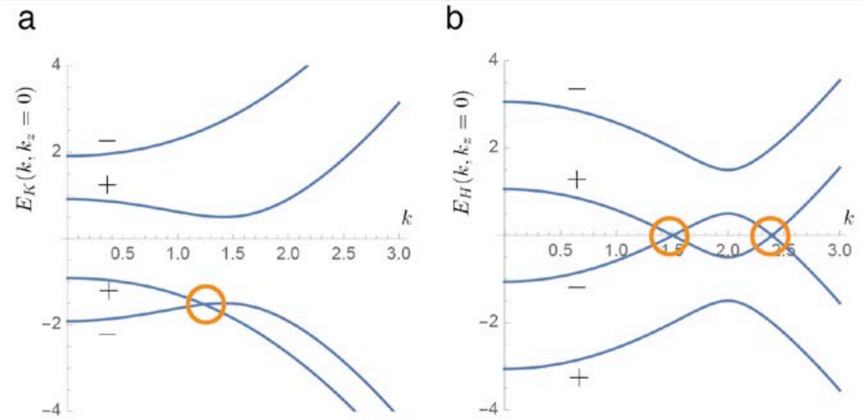

Supplementary Figure 1: ARPES spectra Band structure of the effective *k•p* models. R_z reflection eigenvalues of the bands are obtained from DFT calculations and reflection-protected crossings that correspond to the nodal lines are indicated by orange circles. All bands are nondegenerate and the momentum $\boldsymbol{k} = \sqrt{\boldsymbol{k}_x^2 + \boldsymbol{k}_y^2}$, k_z =0 is measured relative to the high-symmetry points *K* and *H* for panels **a** and **b**, respectively. (**a**) Spectrum of the effective model given by Supplementary Equations 3 and 5 that approximates the low energy bands around the *K* point for parameters *m*=1, μ=0, $\boldsymbol{m'}$=1, $\Delta_{\mathbf{SOC}}$=0.5 and an additional term $\boldsymbol{\Delta'}_{\mathbf{soc}}$ =1.0 proportional to $\tau_0 \otimes \sigma_1$ to break the spectral particle-hole symmetry. (**b**) Spectrum of the effective model given by Supplementary Equations 4 and 6 that approximates the low energy bands around the *K* point for parameters *m*=1, μ=2, $\boldsymbol{m'}$=1, $|M_H|$=0.5.

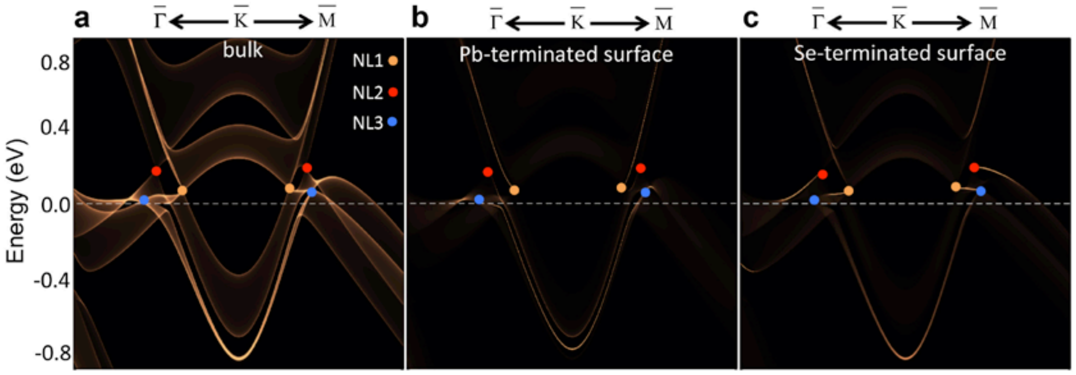

Supplementary Figure 2: Connection of surface bands to bulk nodal lines. (**a**) (001)-projected bulk bands of $PbTaSe_2$. Color balls indicate the location of three nodal lines. (**b**,**c**) Surface-weighted band structure of Pb-terminated and Se-terminated $PbTaSe_2$(001) surface, respectively.

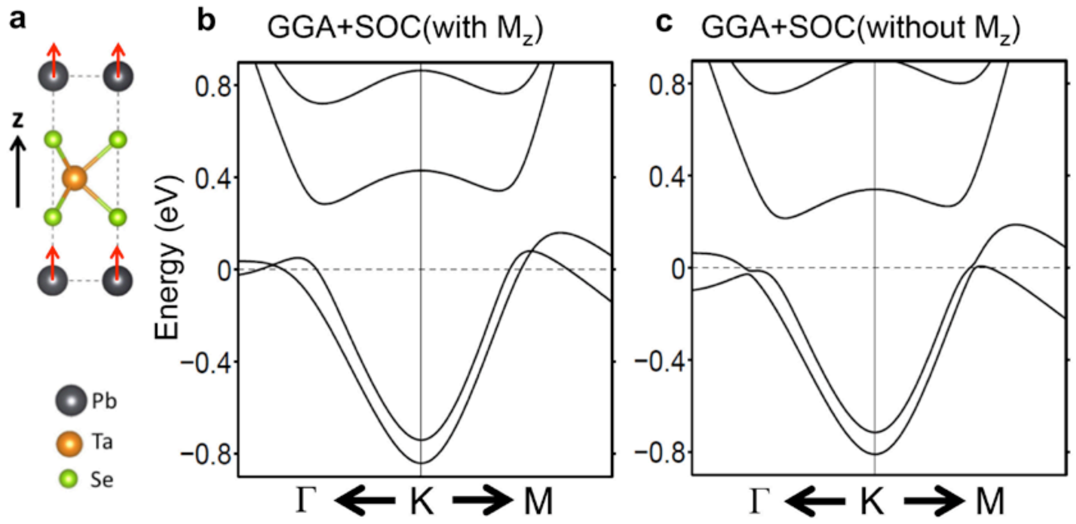

Supplementary Figure 3: Band structure of NL3 with the inclusion of reflection-symmetry breaking perturbation. (**a**) Schematic diagram showing the atomic displacement which breaks the reflection symmetry. (**b**,**c**) Band dispersion of NL3 without/with the inclusion of the reflection-symmetry breaking lattice distortion shown in panel **a**.

Supplementary Note 1: Description of lines nodes in $PbTaSe_2$ by an effective ***k•p*** Hamiltonian

The reflection symmetry R_z that sends z to $-z$ in position space acts on the spin degree of freedom like the third Pauli matrix σ_3. The crystal structure of $PbTaSe_2$ is layered in the z direction, where Pb and Ta layers are alternating. For spinless systems, a consistent choice of the action of R_z is

$$R_z \psi_{\mathrm{Pb}|n_x,n_y,n_z} = \psi_{\mathrm{Pb}|n_x,n_y,-n_z}, \quad R_z \psi_{\mathrm{Ta}|n_x,n_y,n_z} = \psi_{\mathrm{Ta}|n_x,n_y,-n_z-1}, \tag{1}$$

where $\psi_{Pb|n_x,n_y,n_z}$ and $\psi_{\mathrm{Ta}|n_x,n_y,n_z}$ are the single-particle wave functions of an electrons in the Pb and Ta orbitals in the unit cell indexed by the integers n_x, n_y, n_z. For Bloch states in the planes k_z=0 and $k_z=\pi$ the action of R_z is local in momentum space. If ψ_{k_x,k_y,k_z} is a 4-spinor in the space of spin and Pb/Ta orbital degrees of freedom, then R_z has the representations

$$R_z \psi_{kx,ky,0} = i\tau_0 \otimes \sigma_3 \psi_{kx,ky,0}, \quad R_z \psi_{kx,ky,\pi} = i\tau_3 \otimes \sigma_3 \psi_{kx,ky,0}, \tag{2}$$

where the Pauli matrices σ_i act on the spin space and the Pauli matrices τ_i act on the Pb/Ta orbital space.

We now give an effective ***k•p*** Hamiltonian to explain how the line nodes centered around the *K*/*K'* points as well as around the *H*/*H'* points arise. We will focus on *K* and *H*, implying that the Hamiltonian around *K'* and *H'* follows as its time-reversal symmetric conjugate. We observe from the first principal calculations that the dispersions for the Pb and Ta orbitals are particle- and hole-like, respectively, around these high-symmetry points.

In absence of spin-orbit coupling, an effective Hamiltonian for small momenta away from these points can be written as

$$H_K^0(k_x,k_y,k_z) = \left(\frac{k_x^2 + k_y^2}{2m} - \mu \right) \tau_3 \otimes \sigma_0 + m' \tau_1 \otimes \sigma_0, \tag{3}$$

$$H_H^0(k_x,k_y,k_z) = \left(\frac{k_x^2 + k_y^2}{2m} - \mu \right) \tau_3 \otimes \sigma_0 + v k_z \tau_1 \otimes \sigma_0, \tag{4}$$

which, for k_z=0 commutes with the respective representations $i\tau_0 \otimes \sigma_3$ and $i\tau_3 \otimes \sigma_3$ of R_z.

The remarkable difference is that H_H^0 supports a ring-node while H_K^0 simply has two bands that are separated in energy. We are omitting terms proportional to $\tau_0 \otimes \sigma_0$ which are irrelevant for topological properties but breaks the (artificial) particle-hole symmetry of Supplementary Equations. 3 and 4.

We will include spin-orbit coupling terms that are constants to lowest order in $\boldsymbol{k}$. Relevant mass terms are given by

$$H_K^{(\mathrm{soc})}(k_x,k_y,k_z) = \Delta_{\mathrm{soc}}\,\tau_3 \otimes \sigma_3 + k_z V_K, \tag{5}$$

where V_K can be any matrix that anticommutes with $\tau_0 \otimes \sigma_3$, such as $\tau_1 \otimes \sigma_1$, and

$$H_H^{(\mathrm{soc})}(k_x,k_y,k_z) = \Delta_{\mathrm{soc}}\,\tau_3 \otimes \sigma_3 + M_H, \tag{6}$$

where M_H can be any matrix that anticommutes with the kinetic term $\tau_3 \otimes \sigma_0$, but commutes with the mirror symmetry R_z ($i\tau_3 \otimes \sigma_3$)such as $\tau_1 \otimes \sigma_1$.

Let us consider $H_K^0 + H_K^{(\mathrm{soc})}$ for k_z=0. The bands are given by

$$E_K(k_x,k_y,0) = \pm\sqrt{m'^2 + \left(\frac{k_x^2+k_y^2}{2m} - \mu \pm \Delta_{\mathrm{soc}}\right)^2}\ , \tag{7}$$

with uncorrelated signs. The lower and upper pair of these bands are degenerate along a line with $k_x^2 + k_y^2 = 2m\mu$. By introducing a term $\Delta'_{\mathrm{soc}}\,\tau_0 \otimes \sigma_3$ the line degeneracy can be made to appear only in the lower pair of bands, as seen in the DFT band structure. The degeneracy line is protected by the opposite mirror eigenvalues of the two bands that cross.

Let us consider $H_H^0 + H_H^{(\mathrm{soc})}$ for $k_z = \pi$. The bands are given by

$$E_H(k_x,k_y,\pi) = \pm\Delta_{\mathrm{soc}} \pm \sqrt{|M_H|^2 + \left(\frac{k_x^2+k_y^2}{2m} - \mu\right)^2}\ , \tag{8}$$

with uncorrelated signs, where $|M_H|$ are the magnitudes of the eigenvalues of the matrix M_H, which are all assumed to be equal. The middle two bands are degenerate along two lines with $k_x^2 + k_y^2 = 2m(\mu \pm \sqrt{\Delta_{\mathrm{soc}}^2 - |M_H|^2})$. These two nodal lines are protected by the opposite mirror eigenvalues of the crossing bands.

The system as a whole is spinful and time-reversal symmetric, placing it in symmetry class AII. The reflection symmetry R_z commutes with the time-reversal symmetry $\boldsymbol{T}= \boldsymbol{K}\sigma_2$, where $\boldsymbol{K}$ is complex conjugation. This places it in class R_+/AII in the classification of topological fermi surfaces. However, since the disk enclosed by each nodal line is not invariant under time-reversal symmetry (time-reversal maps K to K' and H to H'), the presence of this symmetry does not impose any restrictions on the topology of this system. It should thus be located in the time-reversal breaking class A with reflection symmetry, the Fermi lines of which are in three dimensions protected by a mirror-**Z** topological invariant. In fact, all three nodal lines (one around K and two around H) are topologically protected in that way. They carry a topological invariant n^+ given by the difference the number of occupied bands with R_z eigenvalue $+i$ inside and outside of each ring. The ring around K has n^+=-1 (NL3) and the two rings around H have n^+=+1 (NL1) and n^+=-1 (NL2), respectively. The index n^+ also gives the number of surface states emanating from a nodal line in a slab geometry with periodic boundary conditions in the x and y directions.

Supplementary Note 2: Connection of surface bands to bulk nodal lines

To visualize the connection of surface bands to bulk nodal lines, we plotted out in Supplementary Fig. 2 the surface-weighted band structure of $PbTaSe_2$(001) surface. Supplementary Figure 2a shows the (001)-projected bulk band structure. The location of three bulk nodal lines is marked by color balls. The surface band structure weighted by charge density within the top atomic layer is shown in Supplementary Fig. 2b and c for Pb-terminated and Se-terminated surfaces, respectively. In the Pb-termination case, the surface bands disperse outwards, with respect to $\bar{K}$, from NL1. The surface band connecting to NL2 grazes inwards the edge of lower bulk Dirac cone and merge into the bulk band. The surface band from NL3 disperses inwards with respect to $\bar{K}$, consistent with the "SS_2" band in our ARPES spectrum in Fig. 3, which forms a "drumhead" surface state contour. In contrast, on the Se-terminated surface the surface band connecting to NL2 first moves into the bulk band gap and then fall into the bulk band region. The surface band from NL1 disperses outwards and connects to NL3. Even though the dispersion of the surface bands is sensitive to surface conditions, we can always find a surface band connecting to each nodal lines, indicative of the topological nature of the nodal lines.

Supplementary Note 3: Effect of reflection-symmetry breaking perturbation on the "NL3" line node

Complementary to the discussion on the effect of reflection-symmetry breaking perturbation in the main text, we calculated the band structure around *K* with a perturbation which breaks the reflection symmetry. The Pb atoms are shifted slightly in the vertical direction as schematically shown in Supplementary Fig. 3 and, thus, the unit cell no longer preserves the reflection symmetry with respect to the Ta atomic plane. The resulting band structure shows an energy gap opens at the line node of NL3 on k_z = 0 plane.
